# Specific Gene- and MicroRNA-Expression Pattern Contributes to the Epithelial to Mesenchymal Transition in a Rat Model of Experimental Colitis

**DOI:** 10.1155/2017/5257378

**Published:** 2017-05-10

**Authors:** Éva Boros, Marianna Csatári, Csaba Varga, Balázs Bálint, István Nagy

**Affiliations:** ^1^Institute of Biochemistry, Biological Research Centre of the Hungarian Academy of Sciences, Szeged, Hungary; ^2^Department of Physiology, Anatomy and Neuroscience, University of Szeged, Szeged, Hungary; ^3^Seqomics Biotechnology Ltd., Mórahalom, Hungary

## Abstract

The aim of this study was to determine the gene- and microRNA-expression profile contributing to epithelial to mesenchymal transition in a rat model of experimental colitis. For this, inflammation was induced by injecting 2,4,6-trinitrobenzene sulphonic acid to the colon of male Wistar rats. Samples were taken from both inflamed and uninflamed regions of the same colon, total RNA was isolated, and the mRNA and microRNA expressions were monitored. We have determined that the expression of genes responsible for inducing mesenchymal phenotype, such as Egr1, Fgf2, Fgf7, Jak2, Notch2, Hif1α, Zeb2, Mmp9, Lox, and Vim, was all significantly induced in the inflamed regions of the affected colons while the epithelial marker E-cadherin (Cdh1) was downregulated. In contrast, the expression of microRNAs miR-192, miR-143, miR-375, miR-30a, miR-107, and miR-200b responsible for the regulation of the above mentioned genes was significantly downregulated in inflamed colon. Importantly, we detected moderate induction in the expression of five out of six tested microRNAs in the uninflamed regions. In summary, we identified numerous interacting genes and microRNAs with mutually exclusive expression pattern in inflamed regions of colitis-induced rats. These findings suggest that—among others—an important step in the epithelial to mesenchymal transition in experimental colitis is the dysregulated microRNA expression.

## 1. Introduction

Inflammatory bowel disease (IBD) is a chronic relapsing disorder of the gastrointestinal tract. The two main clinical appearances of IBD are ulcerative colitis (UC) and Crohn's disease (CD): both cause idiopathic, returning inflammation along the digestive system, where inflamed and uninflamed regions sporadically follow each other. Primary symptoms of this lifelong disease are abdominal pain, diarrhoea, and malabsorption which are significantly affecting the quality of life. Chronic inflammation, the main feature of IBD, is also a well-known factor in cancer progression. In line with this, IBD significantly increases the probability of colorectal cancer [1, 2]. A crucial step in cancer development and progression of chronic inflammatory diseases is the epithelial to mesenchymal transition (EMT). During EMT cell-cell connections disintegrate, the expression of extracellular matrix components, such as vimentin and metalloproteases, increases and as a consequence cells migrate to the interstitium. Under physiological conditions, EMT is an essential process of wound healing; however, in the absence of proper control, motile cells move through the basal membrane and cause inflammation [3, 4]. The progression of EMT is regulated by complex molecular mechanisms: transcription factors, such as Snail, Twist, and Zeb, suppress the expression of a well-known epithelial marker E-cadherin (Cdh1). Subsequently, decreased expression of Cdh1 leads to the failure of cell-cell connections; therefore, mesenchymal phenotype emerges. In this process, interrelated signalling pathways, such as Notch and JAK-STAT pathways, also promote the formation of the mesenchymal phenotype [5–9].

Genes known to play role in EMT may be regulated by small, noncoding microRNA (miRNA) molecules, which are directly influencing the translation or degradation of messenger RNAs (mRNAs) by binding to their 3′-UTRs. miRNAs can also affect epigenetic landscape through the inhibition of DNA methyltransferase genes, thereby causing global gene expression changes [10]. The involvement of miRNAs, such as the miR-200 family members (miR-200a, miR-200b, miR-200c, miR-141, and miR-429), regulating EMT has been shown earlier; in addition, their dysregulated expression was described in several oncologic conditions [11, 12]. Furthermore, EMT-associated miRNA/mRNA signatures of clear-cell renal cell carcinoma patients highly correlated with clinical stages of cancer progression, which imply the prognostic potential of combined miRNA/mRNA expression profiling in different EMT-related diseases [13].

Understanding the molecular mechanisms inducing and regulating EMT—such as transcriptional and posttranscriptional modifications—upon chronic intestinal inflammation are critical for understanding the exact pathomechanism of ulcerative colitis and Crohn's disease. In line with this, the aim of this study was to determine the expression patterns of EMT-related genes as well as miRNAs regulating these genes in the inflamed and uninflamed colon regions of the 2,4,6-trinitrobenzene sulphonic acid- (TNBS-) induced rat model of experimental colitis.

## 2. Materials and Methods

### 2.1. In Vivo Rat Model and Sample Collection

**Figure 1:**
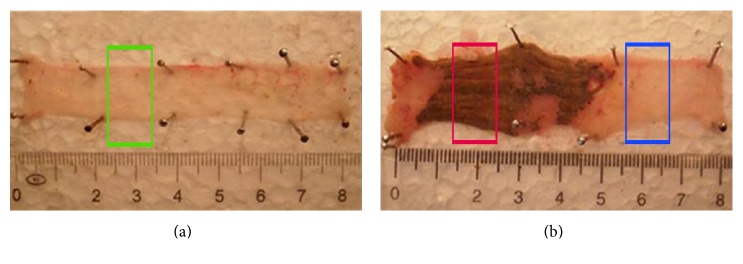
Representative images of noninduced (a) and colitis-induced (b) colons. Green box in (a) indicates control sampling; red and blue boxes in (b) indicate sampling from colitis-induced inflamed and uninflamed regions, respectively.

We used male Wistar rats weighing 180–220 g. All rats had access to water and food ad libitum, except overnight before induction of colitis. The animals were randomly divided into two groups: the first group served as control (*n* = 2; vehicle-treated hence noncolitis induced) and the second group was induced by 2,4,6-trinitrobenzene sulphonic acid (TNBS) (*n* = 6; colitis-induced) based on the method described by Morris et al. [14]. Briefly, TNBS (10 mg in 0.25 ml of 50% ethanol, *w*/*v*) was intracolonically administered in a volume of 0.25 ml, via an 8 cm long plastic catheter under transient ether anaesthesia. 72 hours after the treatment, all animals were sacrificed and ~8 cm of distal colons were removed, opened longitudinally, and gently cleaned of fecal content using ice-cold physiological saline. In the case of the control group, since noninflamed, samples were taken from random colon sections (Figure 1(a)). In case of colitis-induced animals, samples were taken from inflamed colon region as well as from nonadjacent uninflamed region (Figure 1(b)). All samples were kept in TRIzol reagent (Thermo Fisher) at −80°C.

### 2.2. Extraction of Total RNA and Reverse Transcription

Samples from the colon were homogenized in TRIzol reagent by ULTRA-TURRAX T-18 (IKA) instrument as described previously [15]. 0,1 ml of chloroform (Sigma-Aldrich) was added to 0,3 ml homogenized sample with vigorous vortexing. Samples were centrifuged at 13000 rpm for 10 minutes. Total RNA was than extracted from the upper aqueous phase with RNeasy Plus Mini Kit (Qiagen) according to the manufacturer's protocol. The quality and the quantity of the extracted RNAs were determined by TapeStation (Agilent) and Qubit fluorometer (Thermo Fisher). The extracted total RNA samples were only used if the RNA integrity number (RIN) was greater than 7.

Reverse transcription was performed by two different kits, depending on the type of RNA to be reverse transcribed. For mRNA detection, cDNA was synthesized using SuperScript VILO Master Mix (Thermo Fisher) according to the manufacturer's instructions. Briefly, at least 70 ng total RNA was combined with 5X VILO Reaction Mix, 10X SuperScript Enzyme Mix, and nuclease-free water. The reaction was incubated at 25°C for 10 minutes, then at 42°C for 60 minutes, and finally at 85°C for 5 minutes. For miRNA detection, TaqMan MicroRNA Reverse Transcription Kit (Thermo Fisher) was used. Briefly, at least 6 ng total RNA per reaction was mixed with the following components: 100 mM dNTPs, MultiScribe Reverse Transcriptase, 10X Reverse Transcription Buffer, RNase inhibitor, and nuclease-free water. Revers transcription reactions were performed under the following PCR conditions: 30 minutes at 16°C, 30 minutes at 42°C, and 5 minutes at 85°C.

### 2.3. Quantitative Real-Time PCR (QRT-PCR)

miRNA or mRNA expression was measured by real-time PCR using the StepOne PCR Systems (Thermo Fisher). SybrGreen and TaqMan technology-based quantitative real-time PCR was used to quantify the relative abundance of the selected mRNAs. For this, specific exon spanning primer sets were used as listed in Table 1. As controls, we used reaction mixtures without cDNA. Briefly, amplification was carried out in a total volume of 6 *μ*l, containing 1 *μ*l of cDNA, 3 *μ*l of SYBR Select Master Mix (2×), 1,04 *μ*l nuclease-free water, 0,48 *μ*l of forward, and 0,48 *μ*l reverse primer. The QRT-PCR reactions were performed under the following PCR conditions: one cycle of 2 minutes at 50°C and 2 minutes at 95°C, followed by 40 cycles of 15 sec each at 95°C, and 1 minute at 60°C. Detection of nonspecific amplification was carried out by melt curve analysis. TaqMan technology-based reactions were performed in total volume of 4 *μ*l, containing 1 *μ*l cDNA, 2 *μ*l of TaqMan Fast Advanced Master Mix (2×), 0,2 *μ*l TaqMan Gene Expression Assay, and 0,8 *μ*l nuclease-free water. Thermal-cycling conditions were as follows: one cycle of 2 minutes at 50°C and 20 sec at 95°C and followed by 40 cycles of 1 sec at 95°C and 20 sec at 60°C. All of the measurements were performed in duplicate with at least two biological replicates. The ratio of each mRNA relative to the 18S rRNA (Thermo Fisher assay number hs-99999901_s1) was calculated using the 2^−ΔΔCT^ method.

For measurement of miRNA expression levels, TaqMan Universal PCR Master Mix (Thermo Fisher) was used according to the manufacturer's instructions. Briefly, amplification was carried out in a total volume of 15 *μ*l containing the following components: 1 *μ*l miRNA specific cDNA, 0,75 *μ*l TaqMan Small RNA Assay (20×), 7,52 *μ*l TaqMan Universal PCR Master Mix (2×), and 5,73 *μ*l nuclease-free water. Thermal-cycling conditions were as follows: one cycle of 10 minutes at 95°C, 40 cycles of 15 sec at 95°C, and 1 minute at 60°C. The specific miRNA assays were purchased from Thermo Scientific; assay numbers are shown in Table 2. The ratio of each miRNA relative to the endogenous U6 snRNA was calculated using the 2^−ΔΔCT^ method.

### 2.4. Statistical Analysis and Data Representation

Statistical evaluations were performed using the IBM SPSS Statistics program for Windows. Graphs were plotted with GraphPad Prism 6 software. Quantitative data are presented as the mean ± SEM, and the significance of difference between sets of data was determined by one-way analysis of variance (ANOVA) following LSD post hoc test; a *p* value of less than 0.05 was considered significant.

## 3. Results and Discussion

In order to investigate whether EMT is involved in the pathogenesis of chronic colon inflammation, we used the 2,4,6-trinitrobenzene sulfonic acid- (TNBS-) induced rat model of colitis, in which the inflammatory response is due to the generation of transmural oxidative stress and release of proinflammatory mediators [16, 17]. We hypothesized that even though only marked regions of the colon show severe inflammation (Figure 1(b)), proximal, phenotypically not inflamed regions may also be affected. Hence, we decided to take two samples from the colons of the colitis-induced animals: one sample corresponds to the severely inflamed region and the other to noninflamed region, referred as inflamed and uninflamed, respectively; the two regions are being separated by at least 2 cm from each other (Figure 1(b)). Subsequently, we determined the expression profile of selected genes and microRNAs related to EMT.

### 3.1. Induced Expression of Growth Factors at the Site of Induced Colitis

**Figure 2:**
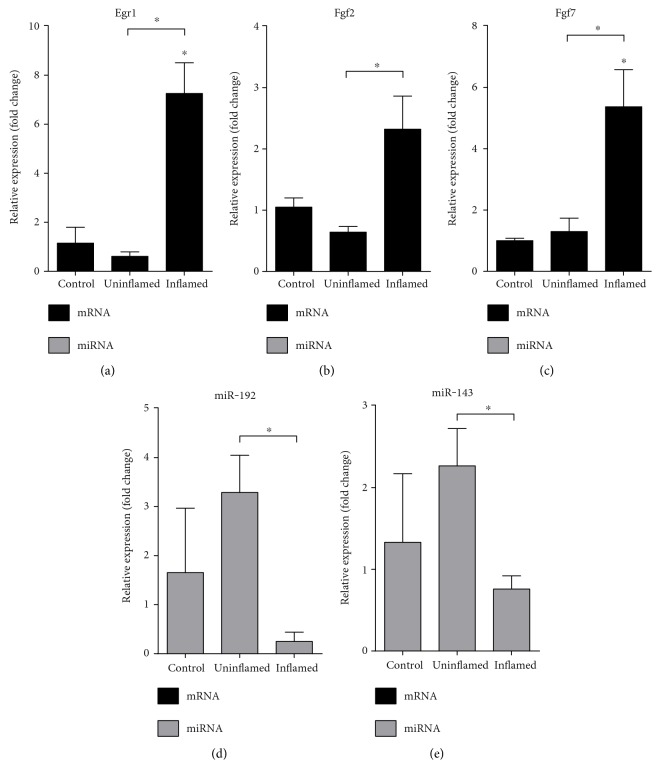
Opposing expression of growth factors and their regulating miRNAs at the site of colon inflammation. The relative gene expression of growth factors Egr1 (a), Fgf2 (b), and Fgf7 (c) shows significant upregulation in inflamed colons as compared to both control and uninflamed colons of colitis-induced animals. In contrast, the expression pattern of both miR-192 (d) and miR-143 (e) shows slight upregulation in uninflamed colons and a significant downregulation in inflamed colons. Data are presented as the mean ± SEM; the significance of differences between sets of data was determined by one-way analysis of variance (ANOVA) using SPSS Statistics; ^∗^*p* < 0.05.

Signalling pathways activating the formation of motile mesenchymal cells are induced by growth factors, such as the early growth response protein 1 (Egr1) and fibroblast growth factors 2 and 7 (Fgf2, Fgf7) [18, 19]. All three genes showed increased expression in inflamed region of the colitis-induced colons as compared to both controls and noninflamed regions (Figures 2(a)–2(c)). It is known that miR-192 regulates the expression of Egr1 and Fgf2, while miR-143 is a known inhibitor of Fgf7 [20]. As compared to controls, both miRNAs are slightly but not significantly induced in uninflamed regions and supressed in inflamed regions. When comparing the expression in uninflamed versus inflamed regions, we detected significant suppression in inflamed regions (Figures 2(d) and 2(e)). The negative correlation between the gene and microRNA expression suggests that the increased mRNA levels of growth factors (Egr1, Fgf2, and Fgf7) may be due to the downregulated expression of their regulating microRNAs (miR-192, miR-143).

### 3.2. JAK/STAT and Notch Signalling Pathways Are Activated upon Inflammation of the Colon

**Figure 3:**
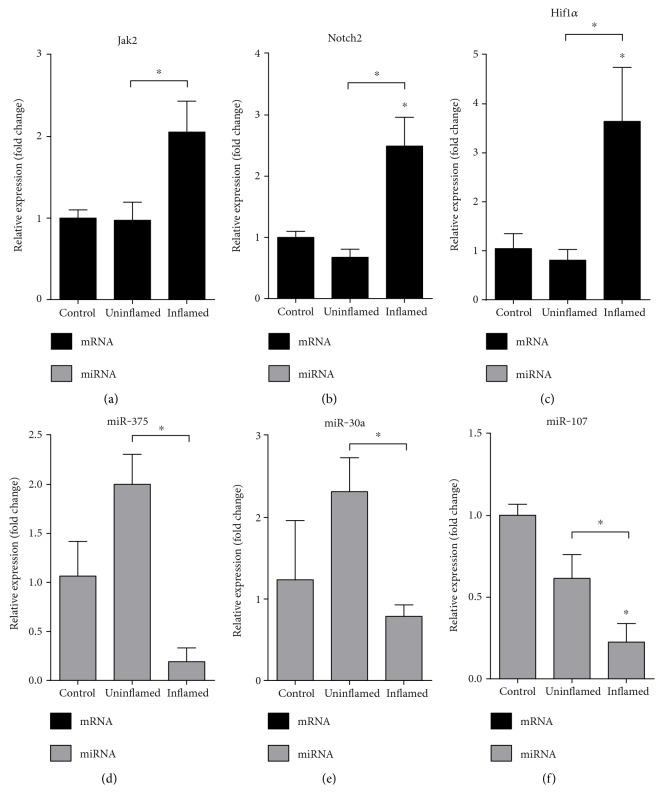
Induced expression of genes regulating proliferation occurs due to decreased expression of miRNAs. The relative gene expression of Jak2 (a), Notch2 (b), and Hif1α (c) shows significant upregulation in inflamed colons as compared to both control and uninflamed colons of colitis-induced animals. In contrast, the expression of miR-375 (d), miR-30a (e), and miR-107 (f) is significantly downregulated in inflamed colons. Data are presented as the mean ± SEM; the significance of differences between sets of data was determined by one-way analysis of variance (ANOVA) using SPSS Statistics; ^∗^*p* < 0.05.

We next monitored the expression profile of key molecules belonging to the JAK/STAT and Notch pathways that play important roles in the regulation of expression of transcription factors associated with EMT [6, 9]. We observed that both Janus kinase 2 (Jak2) and Notch2 were upregulated in the colitis-induced inflamed colon regions (Figures 3(a) and 3(b)). Ding et al. earlier demonstrated that miR-375 directly targets the Jak2 oncogene, thereby regulating cancer cell proliferation [21]. In our model, the amount of miR-375 was significantly decreased in the inflamed region, while elevated in the TNBS treated uninflamed sections (Figure 3(d)). When monitoring the expression of miR-30a, the endogenous inhibitor of Notch2 expression [22], we again detected significant decrease in inflamed samples (Figure 3(e)).

Notch pathway is also induced by hypoxia; therefore, we next examined the expression of a well-known marker of hypoxia and oxidative stress, hypoxia induced factor 1 *α* (Hif1*α*) [6]. mRNA level of Hif1*α* was again significantly induced in inflamed region of the colon (Figure 3(c)). In contrast, the expression of miR-107, a common regulator of Notch2 and Hif1*α* [23, 24], decreased in colitis-induced samples (Figure 3(f)). These data suggest that the absence of inhibition caused by decreased miRNA expression in inflamed regions may shift cell proliferation balancing pathways towards EMT.

### 3.3. Cells Lose Epithelial Nature at the Site of Colon Inflammation

**Figure 4:**
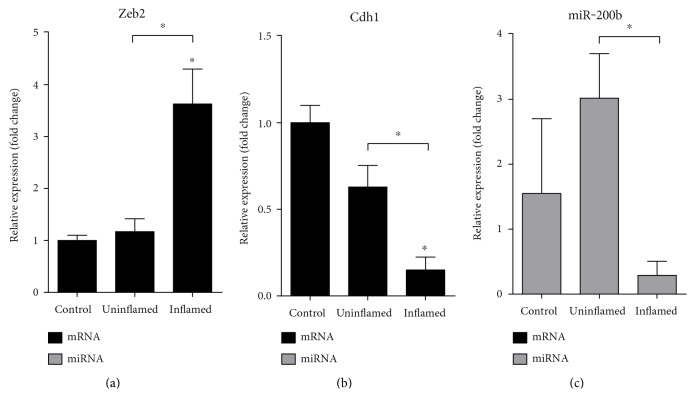
Induced expression of Zeb2 in inflamed colon downregulates the expression of its targets Cdh1 and miR-200b. The relative gene expression of Zeb2 (a) is significantly induced in inflamed colons as compared to both control and uninflamed colons of colitis-induced animals. In contrast, epithelial markers Cdh1 (b) and miR-200b (c) are both significantly downregulated in inflamed colons of colitis-induced animals. Data are presented as the mean ± SEM; the significance of differences between sets of data was determined by one-way analysis of variance (ANOVA) using SPSS Statistics; ^∗^*p* < 0.05.

Transcription factors involved in the progression of EMT are, among others, Snail, Twist, and Zeb [25]. Zeb2 has a fundamental role in the expressional inhibition of E-cadherin coding gene, Cdh1 [26]. This process is under the control of a well described regulatory feedback loop between Zeb2 and miR-200b; importantly, Zeb2 and miR-200b can also inhibit each other [27]. To prove if EMT was indeed induced after colitis induction, we first determined the expression of epithelial marker Cdh1 and its regulator Zeb2. While the mRNA level of Zeb2 was markedly upregulated at the site of inflammation, Cdh1 was significantly downregulated (Figures 4(a) and 4(b)). The expression of miR-200b also inversely correlated with the expression of Zeb2, being markedly downregulated in inflamed regions (Figure 4(c)). It is important to note that miR-192, also known to regulate the expression of Zeb2 [28], exhibits similar expression profile as miR-200b (Figure 2(d)). These data show that the expression of Zeb2 is de-repressed at the site of colonic inflammation due to the decreased expression of its microRNA regulators miR-200b and miR-192. Increased Zeb2 expression, in turn, downregulates epithelial marker Cdh1 expression, and as a result, cells lose their connections as well as apical-basal polarity which all act in favour of epithelial to mesenchymal transition.

### 3.4. Colon Inflammation Is Marked by Gain of Mesenchymal Characteristics

**Figure 5:**
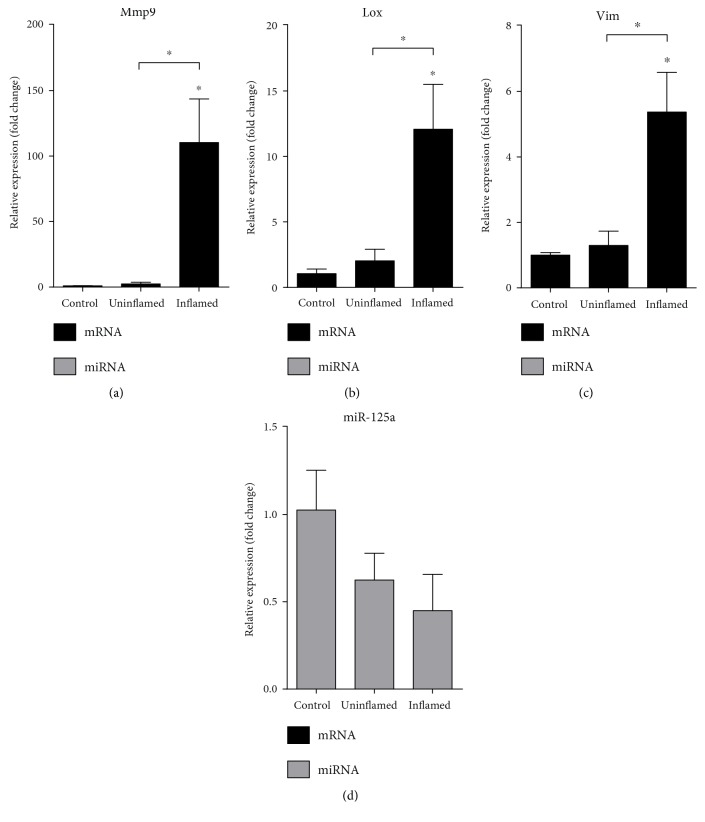
The expression of mesenchymal markers Mmp9, Lox, and Vim is upregulated at the site of colon inflammation. The relative gene expression of Mmp9 (a), Lox (b), and Vim (c) is significantly induced in inflamed colons as compared to both control and uninflamed colons of colitis-induced animals. Conversely, mir-125a (d) shows slight but not significant downregulation in both uninflamed and inflamed colons (*p* = 0.285 and *p* = 0.135, resp.). Data are presented as the mean ± SEM; the significance of differences between sets of data was determined by one-way analysis of variance (ANOVA) using SPSS Statistics; ^∗^*p* < 0.05.

A well-known marker of EMT is the expression of the mesenchymal marker vimentin (Vim) [29]. Furthermore, a prerequisite of cell invasion is the rearrangement of microenvironment by extracellular enzymes, such as matrix metalloproteinase 9 (Mmp9) and lysyl oxidase (Lox) [8]. We have determined that Mmp9, Lox, and Vim all show significantly induced expression in inflamed colon (Figures 5(a)–5(c)) pointing toward gain of mesenchymal phenotype at the site of colon inflammation. Again, miR-125a, miR-30a, and miR-200b regulating Mmp9, Lox, and Vim, respectively [30–32], are all downregulated in inflamed region as compared to uninflamed colon (Figures 3(e), 4(c), and 5(d)). Altogether, these expressional changes confirm that epithelial to mesenchymal transition indeed takes place in colitis induced rats.

## 4. Conclusion

**Figure 6:**
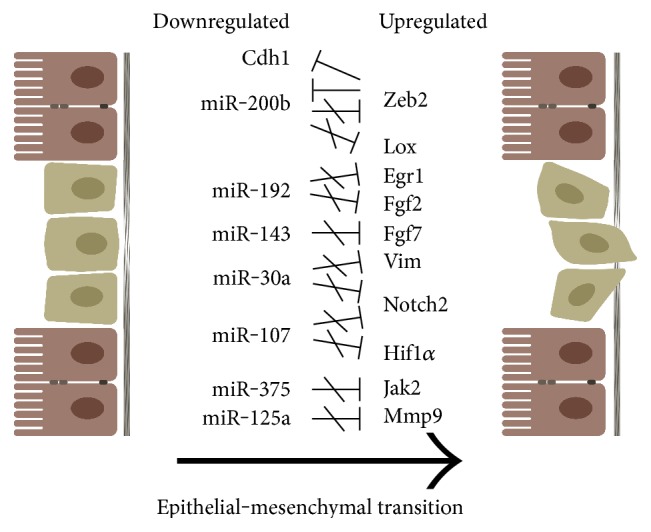
Characteristic features of epithelial to mesenchymal transition at the site of colon inflammation in TNBS-induced rat model of experimental colitis. Because of the downregulated expression, microRNAs are no longer able to regulate the expression of their target genes at the site of colonic inflammation; hence, the expression of genes specific for mesenchymal phenotype increases. Upregulated Zeb2 expression, in turn, downregulates the expression of Cdh1 which further promotes the loss of epithelial phenotype.

Our present study demonstrates that the epithelial to mesenchymal transition is activated during colitis induction in rats. The presented data indicate that, among others, the activation takes place due to the opposed expression profile of genes and their regulating microRNAs at the site of inflammation (Figure 6); while the expression of all tested mesenchymal markers (Egr1, Fgf2, Fgf7, Jak2, Notch2, Hif1*α*, Zeb2, Mmp9, Lox, and Vim) was significantly induced, microRNAs regulating their expression decreased (miR-192, miR-143, miR-375, miR-30a, miR-107, miR-200b, and miR-125a). In parallel, the expression of E-cadherin, a specific marker of epithelial cells, also decreased further supporting the loss of epithelial phenotype at the site of inflammation. These findings shed light onto a microRNA-mediated regulation influencing epithelial to mesenchymal transition in experimental colitis.

## 5. Perspectives

The number of miRNAs shown to be downregulated in IBD is increasing. Elevating the level of these cellular miRNAs using miRNA mimics holds the potential to become a true alternative for therapeutic restoration of physiological pathways lost in IBD. Since miRNAs target multiple genes, restoration of downregulated miRNAs has the potential to have a greater therapeutic effect than drugs with a single protein target [33]. Indeed, recent successful phase IIa studies of miRNA therapy in humans are all in favour of miRNAs being potential therapeutic targets in IBD.

Emerging data also supports the involvement of another class of noncoding RNAs, the long noncoding RNAs, in the epithelial-to-mesenchymal transition. It has recently been shown that HOTAIR (HOX Transcript Antisense Intergenic RNA) mediates a physical interaction between Snail and EZH2 (Enhancer of zeste homolog 2) and thereby influences EMT [34]. In addition, multiple lncRNAs are differentially expressed in IBD and are regulating cellular physiology [35]. Whether lncRNAs may be potential therapeutic targets in IBD remains to be determined.

## Figures and Tables

**Table 1:** SybrGreen primer sets used in QPCR experiments.

Gene	Forward (5′-3′)	Reverse (5′-3′)
Egr1	AACAACCCTACGAGCACCTG	AAAGGGGTTCAGGCCACAAA
Fgf2	GCGACCCACACGTCAAACTA	CCGTGACCGGTAAGTGTTGTA
Fgf7	TGTGGCAATCAAAGGGGTGG	AAGGCCACGAACATTTCCCC
Jak2	AGTGTGCTACAGTGCTGGTC	TTCCTTGTTGCCAGATCCCG
Mmp9	GCCGGGAACGTATCTGGAAA	GGTTGTGGAAACTCACACGC
Notch2	AACTGCACCTCCTCACTTCG	CTCCTCGTTGTTGCATCCCT
Vim	CATGCGGCTGCGAGAAAAAT	GGTCAAGACGTGCCAGAGAA
Zeb2	AAAGCAGTTCCCTTCTGCGA	AGGAGCCCGAGTGTGAAAAG
Hif1*α*	CTCATCCAAGGAGCCTTAACCT	TAACGTTCCAATTCCTGCTGC
Lox	AGGGCGGATGTCAGAGACTA	CATCCAGCAGGTCGTAGTGG
Cdh1	CCACCAGATGACGATACCCG	GAATCACTTCCGGTCTGGCA

**Table 2:** miRNA specific TaqMan microRNA assays.

miRNA	Assay number
miR-125a	002198
miR-192	000491
miR-200b	002251
miR-375	000564
miR-30a	000417
miR-107	000443
miR-143	463509
U6 snRNA	001973
